# The International Collaboration of Pharmacy Journal Editors (ICPJE) formally constituted to foster quality around clinical and social pharmacy practice research publications^*^

**DOI:** 10.1007/s11096-024-01833-8

**Published:** 2024-12-19

**Authors:** F. Alves da Costa, F. Fernandez-Llimos, S. Desselle, I. Arnet, Z. Babar, C. Bond, M. Cordina, V. Garcia Cardenas, M. S. El Hajj, R. Jacobsen, A. V. Law, L. S. Nørgaard, C. Polidori, N. Shcherbakova, D. Stewart, F. Tonin, A. E. Weidmann

**Affiliations:** 1 International Journal of Clinical Pharmacy; Research Institute for Medicines (iMed.ULisboa), Faculty of Pharmacy, University of Lisbon, Lisbon, Portugal; 2 Revista Brasileira de Farmácia Hospitalar e Serviços de Saúde; Laboratory of Pharmacology, Department of Drug Sciences, Faculty of Pharmacy, University of Porto, Porto, Portugal; 3 Research in Social and Administrative Pharmacy; Exploratory Research in Clinical and Social Pharmacy; Touro University California College of Pharmacy, Vallejo, CA USA; 4 Department of Pharmaceutical Sciences, University of Basel, Basel, Switzerland; 5 Journal of Pharmaceutical Policy and Practice, University of Huddersfield, Huddersfield, UK; 6 College of Pharmacy, QU Health, Qatar University, Doha, Qatar; 7 University of Aberdeen, Aberdeen, UK; 8 Department of Clinical Pharmacology and Therapeutics, WHO Collaborating Centre for Health Professionals Education and Research, University of Malta, Malta, Msida Malta; 9 Research in Social and Administrative Pharmacy, University of Granada Faculty of Pharmacy, Granada, Spain; 10 Exploratory Research in Clinical and Social Pharmacy; College of Pharmacy, QU Health, Qatar University, Doha, Qatar; 11 Exploratory Research in Clinical and Social Pharmacy, Department of Pharmacy, University of Copenhagen, Copenhagen, Denmark; 12 Research in Social and Administrative Pharmacy; Western University of Health Sciences, Pomona, California USA; 13 Research in Social and Administrative Pharmacy; Department of Pharmacy, University of Copenhagen, Copenhagen, Denmark; 14 European Journal of Hospital Pharmacy; Experimental medicine and Public health, University of Camerino, Camerino, Italy; 15 Western New England University, Springfield, Massachusetts USA; 16 International Journal of Clinical Pharmacy, Robert Gordon University, Aberdeen, UK; 17 Royal College of Surgeons in Ireland, Dublin, Ireland; 18 Exploratory Research in Clinical and Social Pharmacy; ESTeSL-Escola Superior de Tecnologia da Saúde, Instituto Politécnico de Lisboa, Lisbon, Portugal; 19 International Journal of Clinical Pharmacy; European Journal of Hospital Pharmacy; Faculty for Chemistry and Pharmacy; University of Innsbruck, Innsbruck, Austria

The Granada statements were a result of the need to strengthen clinical, social and administrative pharmacy practice as an area of knowledge that translates into practice, research and policy. As a response, a group of clinical and social pharmacy practice journal editors launched an initiative in Granada in 2023, to discuss ways to improve the quality of publications in this area, which culminated in the Granada statements. Eighteen statements were developed, clustered into six main domains: (1) the appropriate use of terminology; (2) developing impactful abstracts; (3) having the required peer reviews; (4) journal scattering; (5) more effective and wiser use of journal and article performance metrics; and (6) authors’ selection of the most appropriate pharmacy practice journal to submit their work. The full Granada statements have been published in 14 journals [1–14]. These pioneering statements are rooted in similar endeavors undertaken by scholars in other health professions groups, fostering the concept of interdisciplinary consensus and advancing the scientific paradigm [15, 16].

As next step, the chief-editors, handling editors, publishers from the same journals met again in June 2024, this time in Basel, Switzerland, where a formal group name was adopted—the International Collaboration of Pharmacy Journal Editors (ICPJE) following a consensus approach to elect a name that best reflected the group scope, mission and vision. The ICPJE was born.

During the meeting in Basel, the group discussed current issues relating to raising the quality of publications which among other things reflected a need to abate the discipline’s need to re-evaluate itself though papers examining the importance of pharmacy by other stakeholders. These were not cited by papers outside or even within pharmacy, which has been demonstrated by a more holistic examination of drivers of citations through original research [17]. The findings of this study highlight four main factors associated with citations, namely the number of references, the year of publication, social media mentions and the topic area of research, namely pharmacy services and medication adherence. In the context of the discussion, it was emphasized that several previous studies across various disciplines including medical specialties, nursing and other allied healthcare professions have reported diverse findings. While some publications have concurred with the impact of social media highlighting its role in increasing visibility [18], most publications have found the nature of the topic and the methodology employed to be highly relevant factors [19]. This corroborates with the findings of Shcherbakova et al. and others who also pointed out to the relevance of the journal reputation [17, 20]. Furthermore it washighlighted that the COVID-19 pandemic was found to boost citations [20]. Another important determinant is innovation and multicentre or multidisciplinary studies. Some of the aforementioned studies have identified the number of references as a success factor, however in our view, this is mostly indicative of how comprehensive a literature review is [17, 18].

The group did an analysis of the number of Medical Subject Headings (MeSH) used for indexation of clinical and social pharmacy practice articles compared to those in clinical medicine and similar areas and found a significantly lower number of terms. Furthermore, with the full implementation of the automatic indexation by the National Library of Medicine in 2022, this aspect was heightened [21]. It was considered that an essential area that the ICPJE should focus their efforts is to promote the standardization of terms used in pharmacy practice articles, which can be achieved for instance by promoting use of preferred terms to describe systems of care in pharmacy so would help focus searches by researchers and maximize the likelihood of important papers in pharmacy being found. Likewise, the ICPJE will help prospective authors utilize MeSH terms in article titles and abstracts so as to coalesce our efforts in raising this visibility and ameliorating ambiguity around terms not fully recognized by scholars, particularly those outside the discipline.

Whilst in Basel, those present reflected on the accessibility of the Granada statements if they were read without the underpinning justification included in the original paper. It was concluded that each statement needed to be accompanied by a few explanatory sentences, describing the underlying rationale and targeted at the audience for whom the statement was most relevant, i.e., publishers, editors, reviewers and most importantly authors. It was therefore considered crucial to include a wider audience in the revision of the statements and descriptions, embracing the concept of co-creation [22]. To achieve this, it was agreed that three subgroups should be convened, namely one tasked with composing short explanatory sentences to accompany each Granada statement. The second group was tasked with proposing a methodology to create an Early Career Researcher Advisory Board (ECRAB), defining their tasks and duties. The ECRAB will include authors and reviewers from different pharmacy practice journals and will act as a sounding board for the ICPJE, with an initial task to comment on short explanatory statements to accompany the Granada statements and eventually support any rewording needed. Similar initiatives have been proposed by the World Health Organization (WHO) Regional Office for Europe, with the creation of the Youth4Healthspecial initiative, which aims to amplify and embed youth voices and perspectives into all areas of its work (https://www.who.int/europe/initiatives/youth4health). The ICPJE truly believes that this ECRAB has enormous potential to contribute to the external visibility and promotion of clinical and social pharmacy practice research paradigm. The third group convened will focus on embedding the statements into university curricula and part of their duties will be to create a methodology to engage the Higher Education Institutions; the ultimate goal is to increase awareness of the statements and influence their use starting at undergraduate level. Even though the remit of the ICJPE expands way beyond Europe, the recent revision of the European Directive on minimum training requirements for pharmacists (and other healthcare workers) [23] may be an excellent opportunity to ensure adequate knowledge and skills of scientific writing within the context of some of the new compulsory topics, such as pharmaceutical care, clinical pharmacy and public health, as a means to contribute to disseminate and promote knowledge and thus influence policy and practice.

In summary, the ICPJE was constituted from an initial group that met previously in Granada to advance the visibility and quality of research in clinical, social and administrative pharmacy practice. Even prior to its formal naming, the group has made some progress in the past couple of years, although it recognizes the need to consolidate its work. The group is dedicated toward strengthening clinical, social and administrative pharmacy practice, not only as a discipline, but the entire profession, including the patients served by its clinicians and researchers. The ICPJE was founded by a select group of journals but is an open group to any other journal in the field. Each journal is represented by a varied group of individuals, including the editors and publishing companies, making it a dynamic group. The ICPJE will be reaching out to various stakeholders seeking collaboration and insights from fellow scholars and practitioners throughout the world, but also across other disciplines, to help see its goals come to fruition and increase its external validity.
